# Supplementary material for: The Fate of Glutamine in Human Metabolism. The Interplay with Glucose in Proliferating Cells
Source: Metabolites. 2019 Apr 26;9(5):81. doi: 10.3390/metabo9050081 (PMC6571637; doi:10.3390/metabo9050081)
Supplement: Supplementary file 1 [file metabolites-09-00081-s001.pdf]

metabolites

MDPI

*Supplementary Materials 1:* Others solutions for pyruvate synthesis from glutamine. Multiplicity of solutions.

The solution obtained with C2M2N for the maximum rate of pyruvate synthesis from glutamine requires the maximum ATP synthesis flux. In the absence of this constraint, the emerging solution (with absolute fluxes minimization) is quite different (Figure S3c). Indeed, in this solution, pyruvate is synthesized through two pathways. The glutamine derived AKG engages in a TCA cycle in both the reducing (flux = 0.61) and the oxidizing (flux = 0.39) pathways. The reducing pathway gives 0.61 citrate, which are split by ATP citrate lyase (CL) to give OAAc and acetyl-CoA ending to 0.077 palmitate. OAAc generates PEPc (PEPCK1) and then 0.61 pyruvate by PK. The 0.39 oxidizing pathway gives MALm which goes out to give MALc and then 0.39 pyruvate through ME1. There is also a 0.15 cycle involving MDH2 and ME1 which transforms 0.15 NADHm in 0.15 NADPHc to complete the 1.078 NADPHc (IDH1 and ME1) necessary for the synthesis of 0.077 palmitate. In this scenario, no ATP is synthesized and the reductive power of glutamine is consumed in citrate and palmitate synthesis. The mitochondrial production of NADH is low as shown by the low activity of RC1 (0.084) as compared with 3.0 in the previous solution allowing a high ATP synthesis flux.

Using MitoCore, we obtained a slightly different result with 1.41 pyruvate from glutamine and no ATP. In this solution, a great part of pyruvate is obtained with the successive syntheses of serine, glycine, alanine, and then pyruvate. In this pathway, there is a high activity of monoamine oxidase specific of the central nervous system and of diabetic myocardium and a high activity of a mitochondrial lactate dehydrogenase, which is rather unexpected in mammalian cells. Reducing mitochondrial lactate dehydrogenase (R_LDH_Dm_MitoCore) activity to zero gives a similar solution as the solution of C2M2N with no ATP synthesis above and a null activity of RC1 indicating a fine balance of oxidative and reductive glutaminolysis (IDH1 = −0.1 and IDH2 = −0.495) in this case. A maximal ATP synthesis flux of 10.9 can be obtained when maintaining the maximal production of 1 pyruvate for 1 glutamine, which is comparable with the yields obtained with C2M2N. The activity of the respiratory chain is also the same as in C2M2N (R_CI = 3, R_CII = 1, and R_CIII = 4, MitoCore notations). However with MitoCore, pyruvate is made through three different pathways. Glutamine is first transformed in AKGm, then in MALm (flux = 1). From there, three fluxes will synthesize cytosolic pyruvate. The main one (flux = 0.8) goes through mitochondrial malic enzyme (ME2) giving PYRm released from mitochondria through an alanine cycle catalysed by the cytosolic and mitochondrial operation of ALATm and ALATc (alanine aminotransferase or glutamate-pyruvate aminotransferase, equivalent to GOT1 and 2 with ALA in place of ASP and PYR in place of OAA, not represented in Figure S3d). A second pathway (flux = 0.1) goes through MDH2, PEPCK2, PEP/Citrate exchanger and then pyruvate kinase. In the last pathway (flux = 0.1) MALm goes out with malate/phosphate exchanger and gives pyruvate through cytosolic malic enzyme (ME1).

These examples show the diversity of solutions, which can be explored through flux variability analysis (FVA). Furthermore, with C2M2N, but not so easily with MitoCore, we can analyse each solution in terms of separate pathways and transporting cycles, in fact, elementary flux modes (EFMs). It is worth noting that we can represent the MitoCore solution on the C2M2N network with some adaptations.

*Supplementary Materials 2* Decomposition of the metabolic network of Figure 8c in elementary flux modes.

The production of biomass is in blue with XTP, SER, and PYR produced by the glycolysis. The other metabolites of the biomass (ASP, GLUT, GLN, ARG, and PAL) are produced through citrate synthesis in mitochondria fed by pyruvate carboxylase. This is not so usual except for palmitate synthesis. The output flux of citrate is 1.7 which split in 0.88 for palmitate synthesis and 0.88 OAA precursor of the 0.7 ASP. The rest of citrate (0.8) is metabolized through IDH1 (giving a great part of

the NADPH necessary for palmitate synthesis) to give AKG and GLUT (GOT1), and GLN (GS1). The rest of glutamate re-enter the mitochondria to make a minute amount of ARG.

These fluxes are completed by several cycles necessary for the exchanges between mitochondria and cytosol. These cycles share some reactions so that it is not easy to decompose all the fluxes in a sum of cycles. Furthermore, the decomposition is not necessarily unique. One decomposition is the following: The two cycles composing the MAS with a 2.7 flux; a AKGm-GLUTm-GLUTc cycle involving GOT2 (-Glud1) (-T9) T4 with a 1.33 flux. In this cycle, the aspartate and arginine NH3 are incorporated by (-Glud1). Then, we have a 1.027 cycle GLUTc-GLUTm-AKGm-AKGc with T4 and T2. Then, we have a 0.391 citrate cycle: CITm-CITc-AKGc-GLUTc-GLUTm-AKGm through (-IDH3) T1 IDH1 T4 GOT2. All these cycles superimpose to give the flux values (rounded values) indicated on Figure 8c.

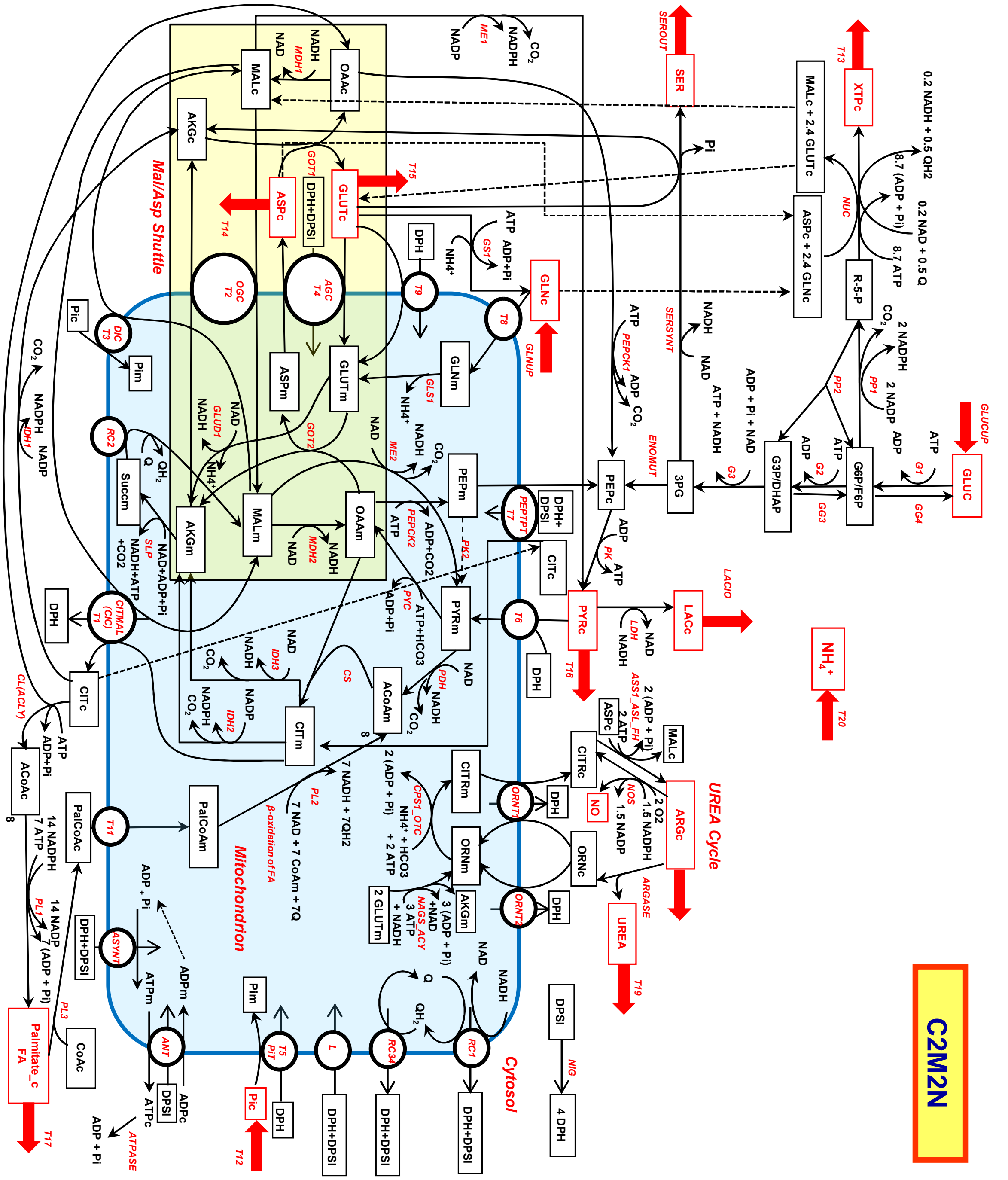

**Figure S1.** Identical to Figure 1 in the main text

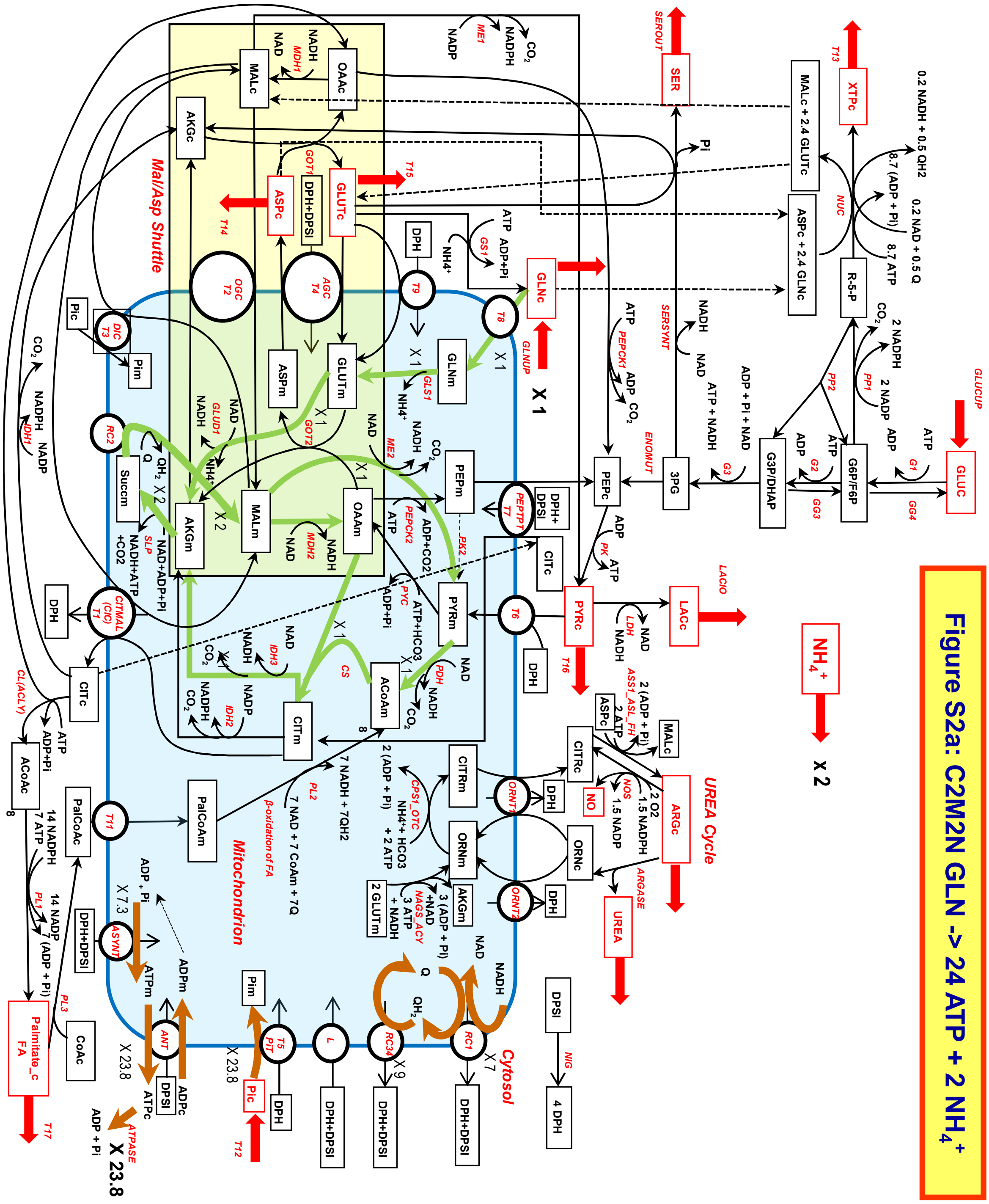


**Figure S2a.** ATP production from glutamine. Green arrows represent the main pathway (flux = 1). Brown arrows depict OXPHOS. A complete TCA cycle is fed by α-ketoglutarate (AKG).

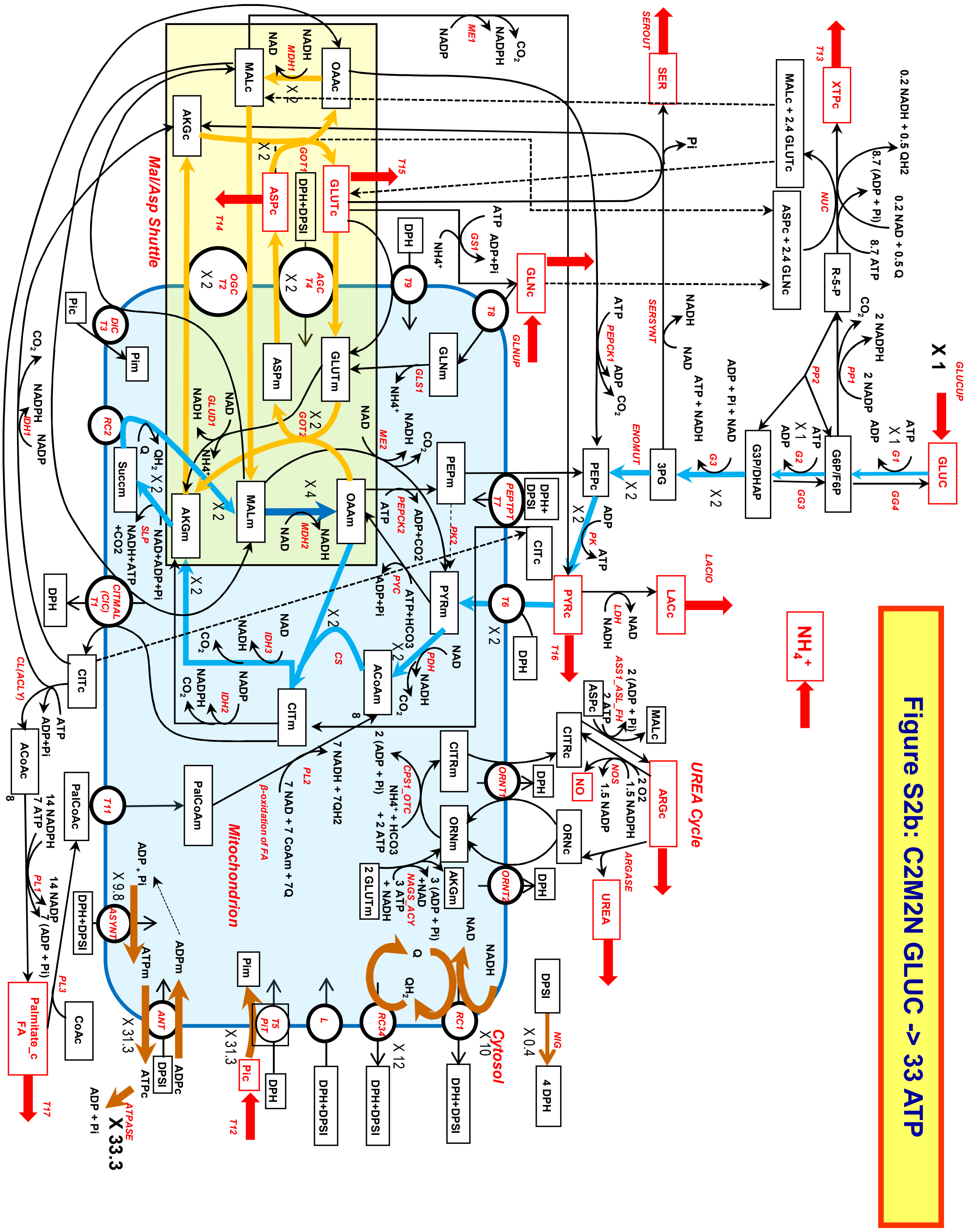


**Figure S2b.** ATP production from glucose. Blue arrows represent the main pathway (flux = 2). Yellow arrows correspond to the malate–aspartate shuttle (flux = 2) to reoxidize glycolytic NADHc. Dark blue arrow means both blue and yellow pathways. Brown arrows depict oxidative phosphorylation (OXPHOS).

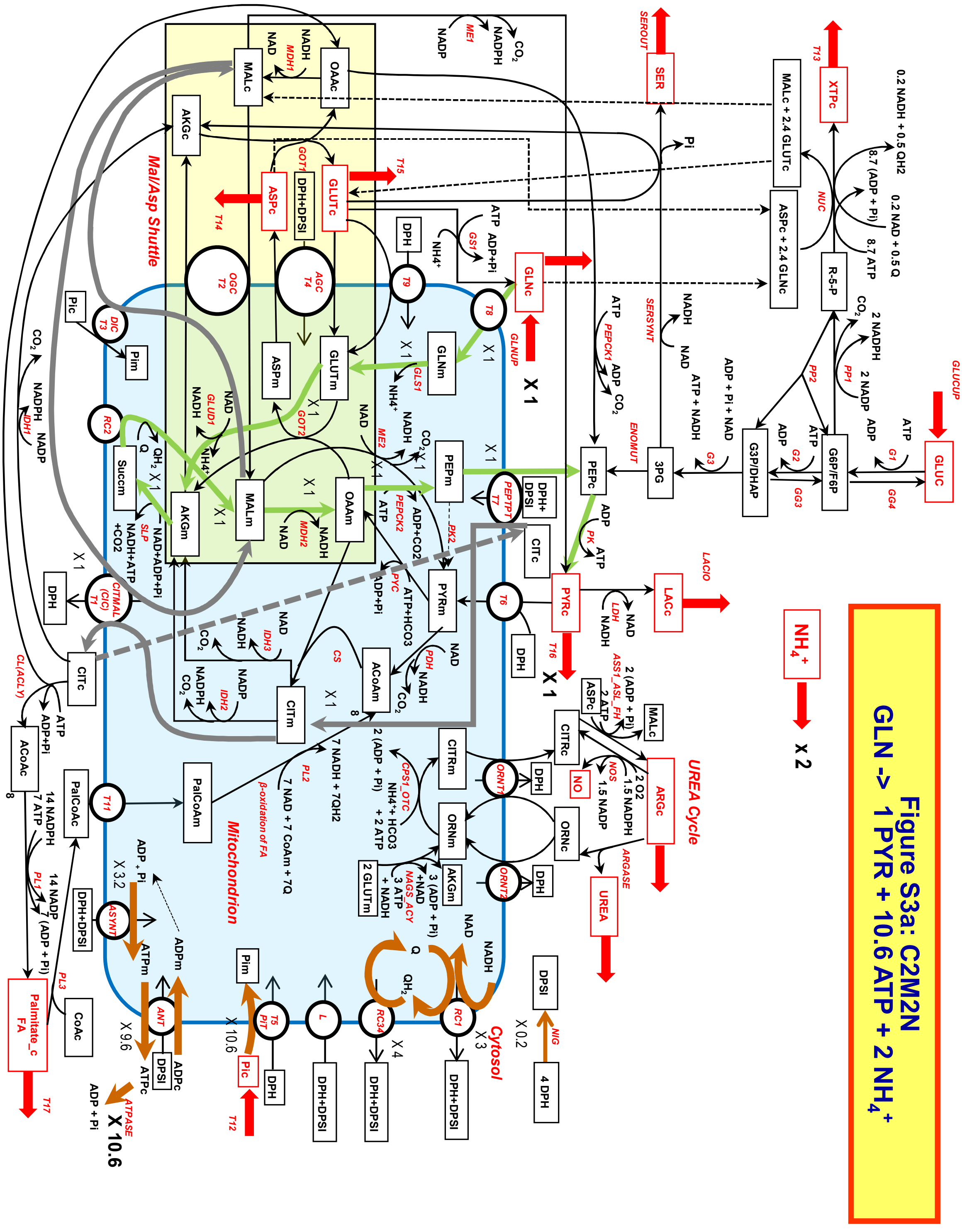


**Figure S3a.** Pyruvate synthesis from glutamine with ATP production. Green arrows represent the main pathway (flux = 1). Brown arrows depict OXPHOS. Grey arrows depict a malate and citrate cycle to allow PEP output from mitochondria.

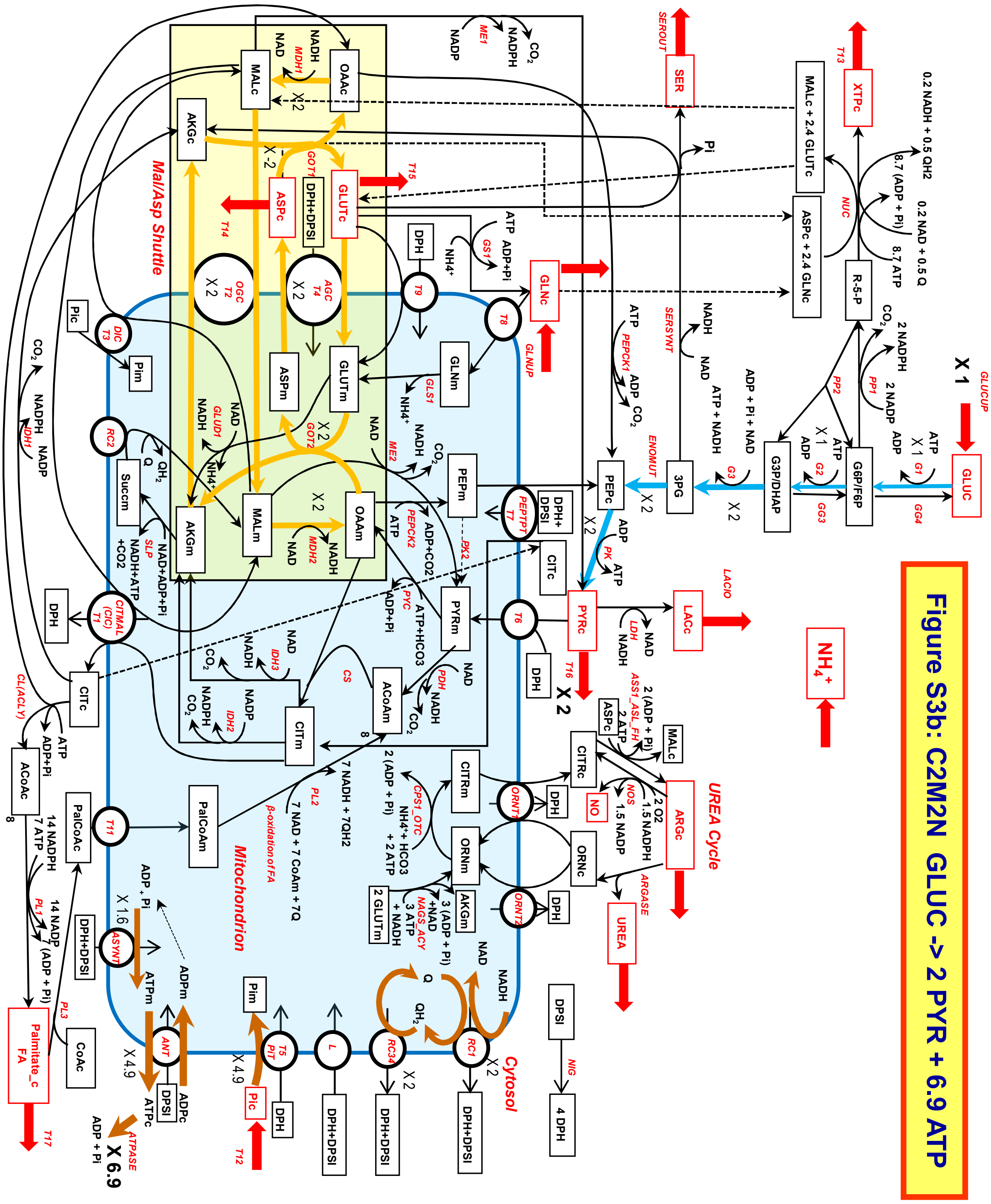


**Figure S3b.** Pyruvate synthesis from glucose. Blue arrows represent the main pathway (flux = 2). Yellow arrows correspond to the malate–aspartate shuttle (flux = 2) to reoxidize glycolytic NADHc. Brown arrows depict OXPHOS.

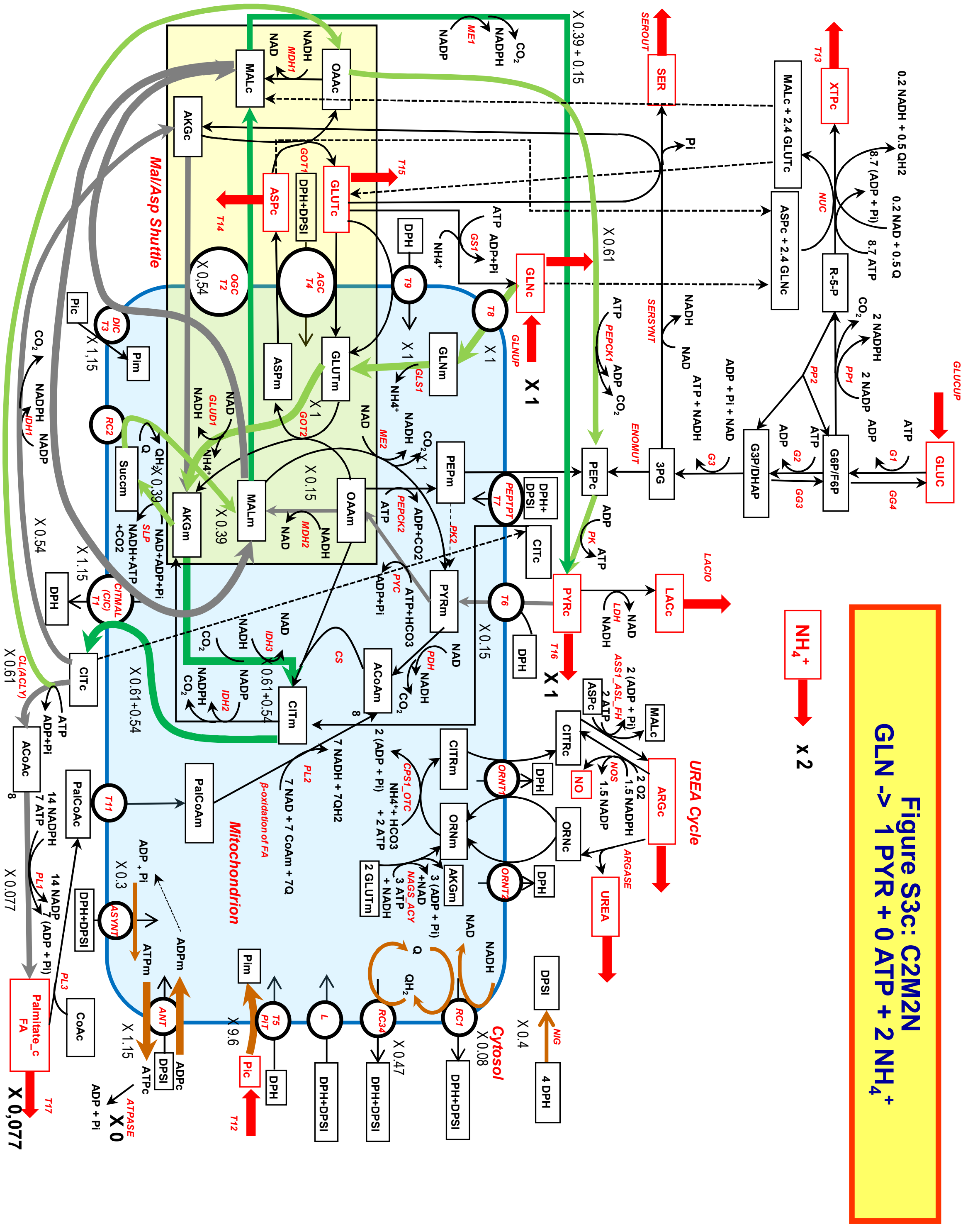


**Figure S3c.** Pyruvate synthesis from glutamine without ATP production but with palmitate synthesis. Green arrows represent the main entry pathway (flux = 1) of glutamine GLS1 and GLUD1). The pyruvate synthesis is in green and the transporting cycles in gray. Pyruvate is synthesized by two pathways: A reducing pathway through IDH3 (dark green arrows = light green + gray fluxes) and an oxidizing pathway through MAL and ME1. Brown arrows depict OXPHOS. Note the low RC1 activity reflecting a low NADHm synthesis. The reducing power of glutamine is transformed in fatty acids (palmitate). See the Supplementary Materials for a full description of this network.

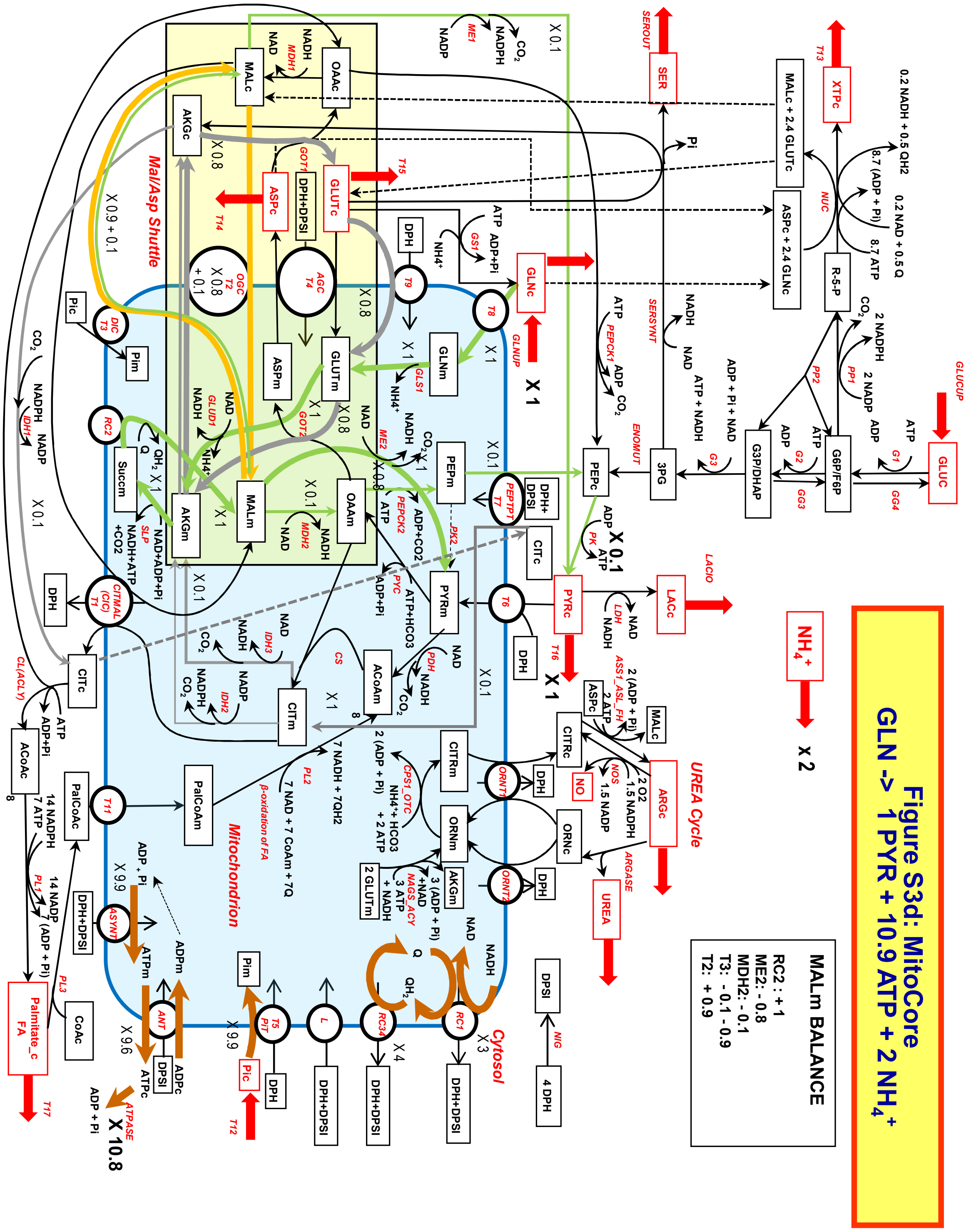


**Figure S3d.** Pyruvate synthesis from glutamine with ATP production using MitoCore. Green arrows represent the three pathways of pyruvate synthesis from glutamine (total flux = 1). The main flux (thick green arrows) goes through ME2 and an alanine transporter not represented here. Two other fluxes (equal to 0.1 each; thin green arrows) go, one through MDH2, PEPCK2, T7, and PK; the other one goes through T3 and ME1. Brown arrows depict OXPHOS. Thin gray arrows (flux = 0.1) depict a citrate/AKG cycle for release of PEPm in cytosol and thick gray arrows (flux = 0.8) depict a AKG/glutamate cycle contributing to alanine transport in cytosol. The yellow arrows depict a malate cycle for AKG transport in the gray AKG/glutamate cycle above.

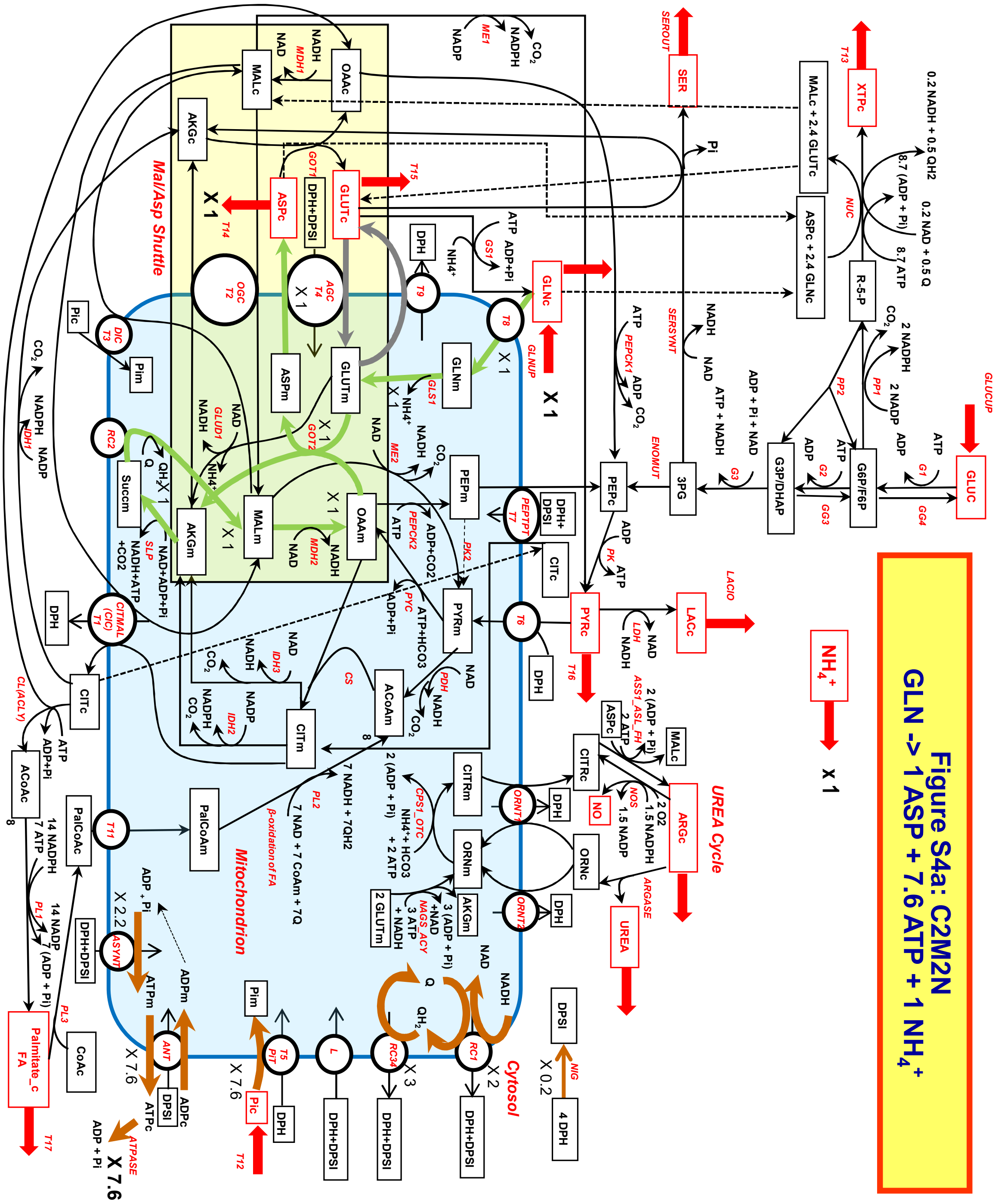


**Figure S4a.** Aspartate synthesis from glutamine. Green arrows represent the main pathway (flux = 1). Gray arrows represent a glutamate cycle to output aspartate. Brown arrows depict OXPHOS.

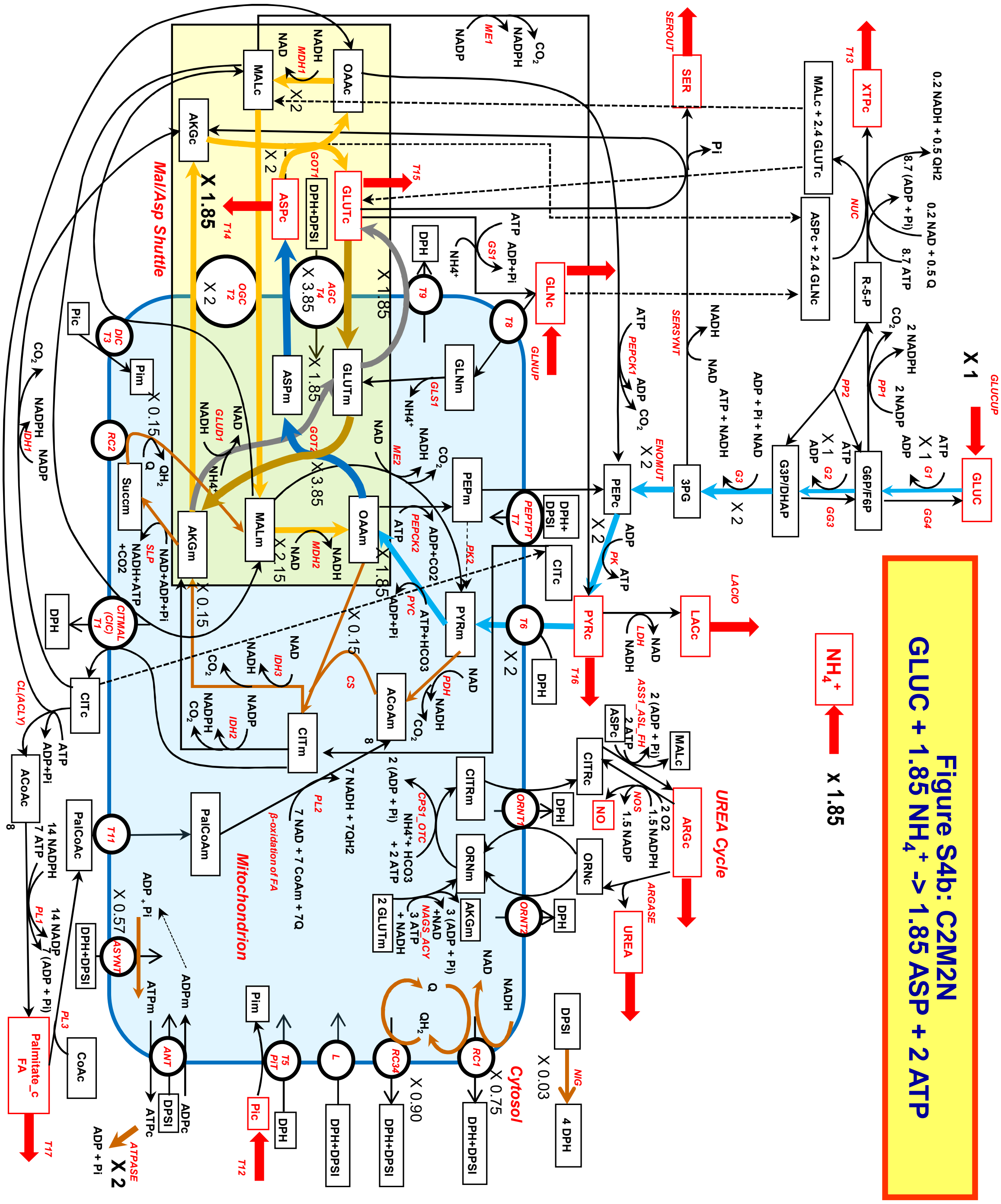


**Figure S4b.** Aspartate synthesis from glucose with production of 2 ATP. Blue and gray arrows represent the aspartate synthesis and output (glutamate cycle) pathways (flux = 1.85). Yellow arrows correspond to the malate–aspartate shuttle (flux = 2) to reoxidize glycolytic NADHc. Dark blue and yellow arrows correspond to mixed pathways (MAS with blue or gray pathway). Pathway in brown arrows (TCA cycle = 0.15 and OXPHOS) are necessary for ATPm synthesis for PYC.

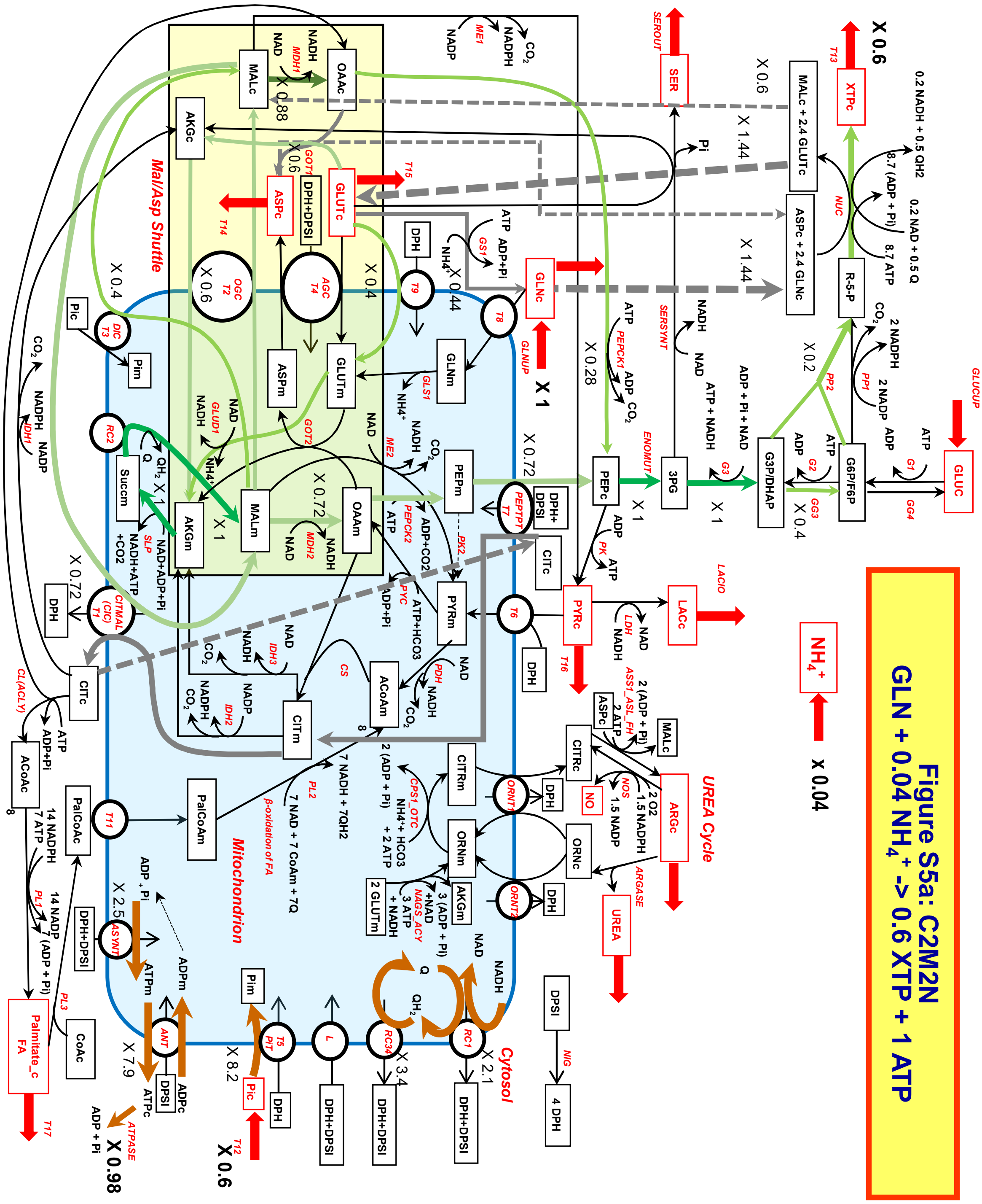


**Figure S5a.** Nucleoside synthesis from glutamine. Green arrows represent the main pathway. Brown arrows depict OXPHOS. Gray arrows represent a citrate cycle necessary to export PEP from mitochondria.

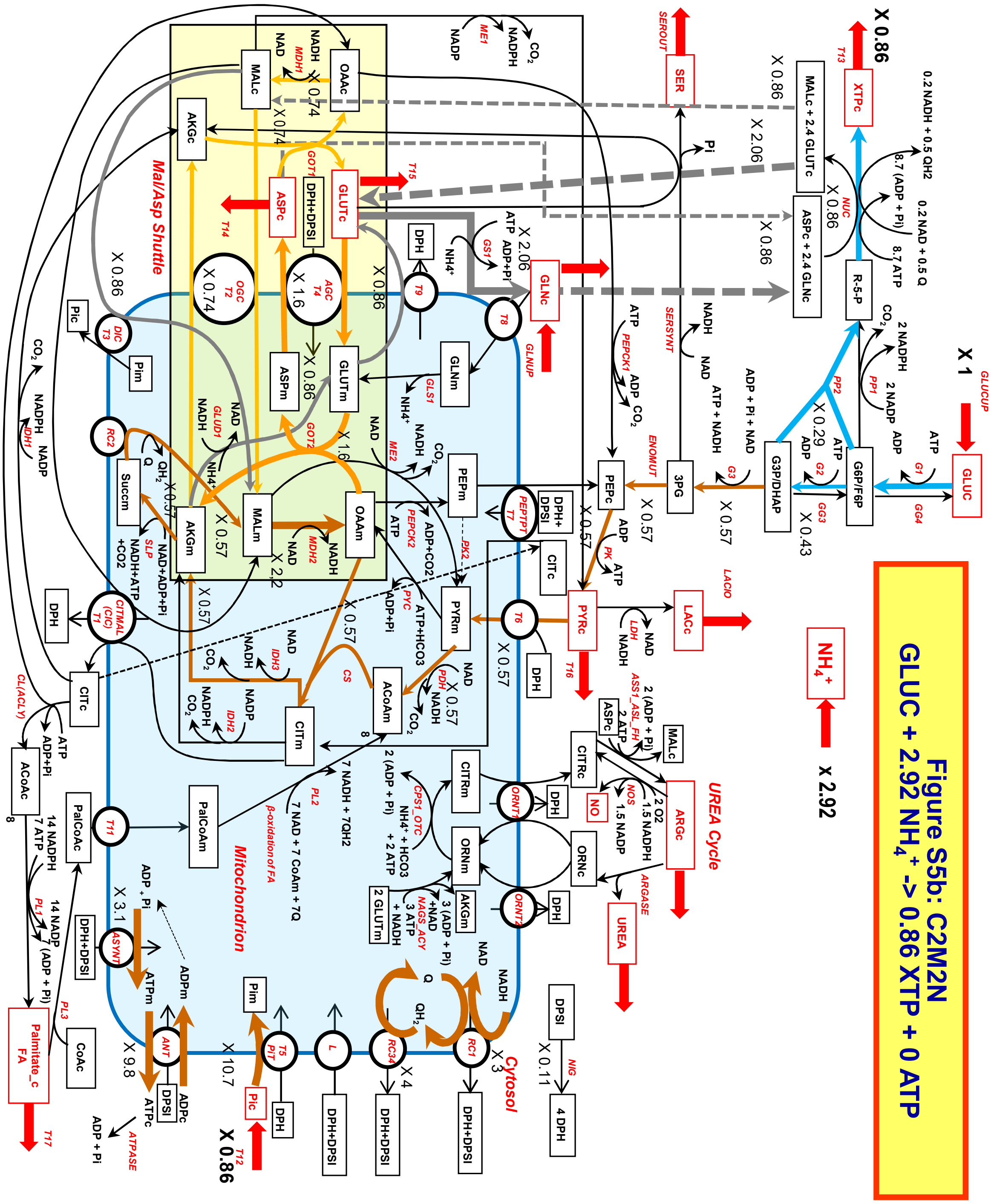


**Figure S5b.** Nucleoside synthesis from glucose. Blue arrows represent the main pathway. Yellow arrows correspond to the malate–aspartate shuttle (flux = 2) to reoxidize glycolytic NADHc. Pathway in brown arrows (end of glycolysis + TCA cycle = 0.57 and OXPHOS) are entirely used for energy production (ATP necessary for nucleotides synthesis (NUC)).

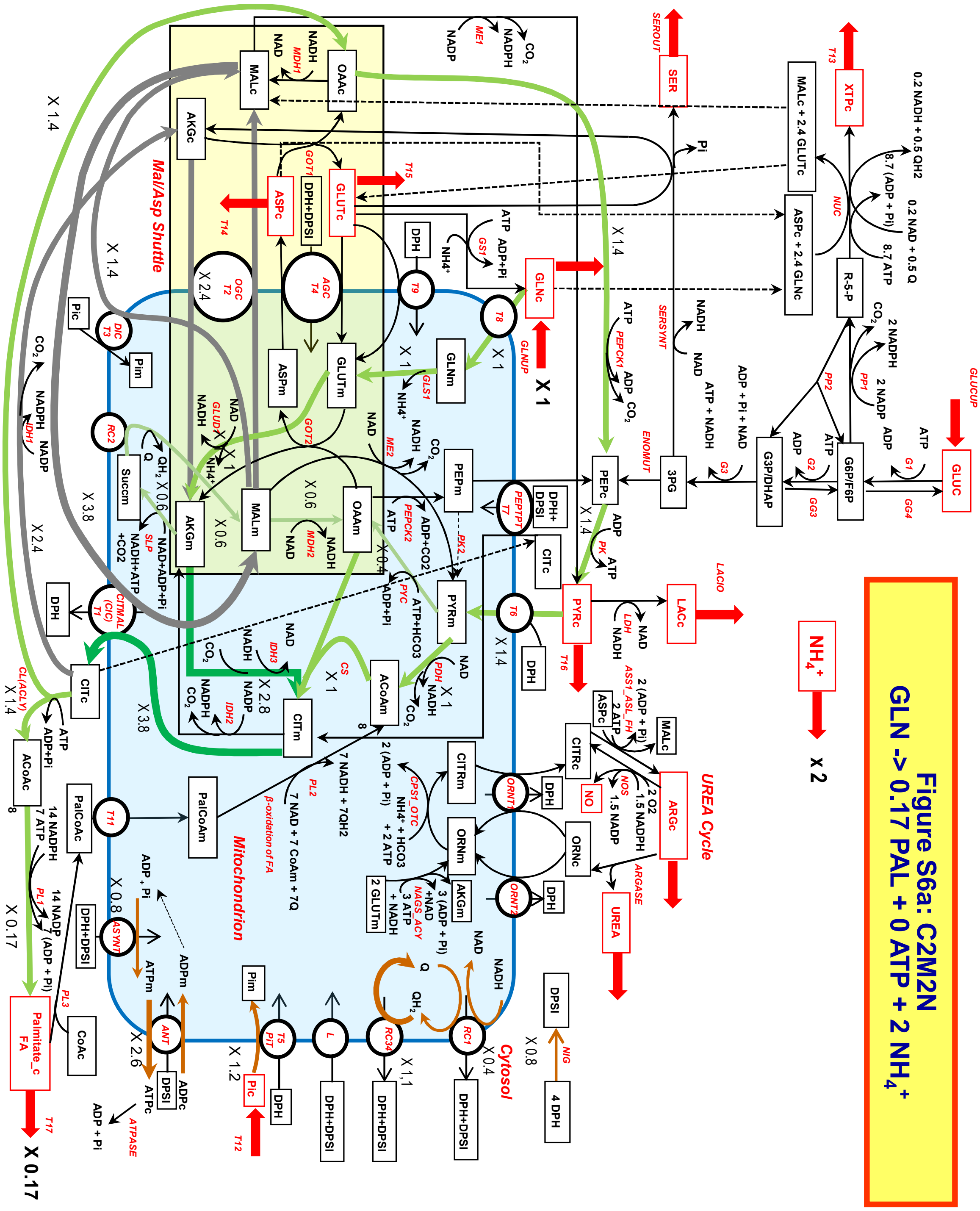


**Figure S6a.** Palmitate synthesis from glutamine. Green and gray arrows represent the main pathway. The glutamine-derived AKG is split in a reductive pathway (reversion of IDH3 in dark green) and an oxidative pathway (light green). There is a cycle involving –IDH3 (dark green) and IDH1 (gray) to transform NADHm in NADPHc for fatty acids synthesis. Brown arrows depict OXPHOS.

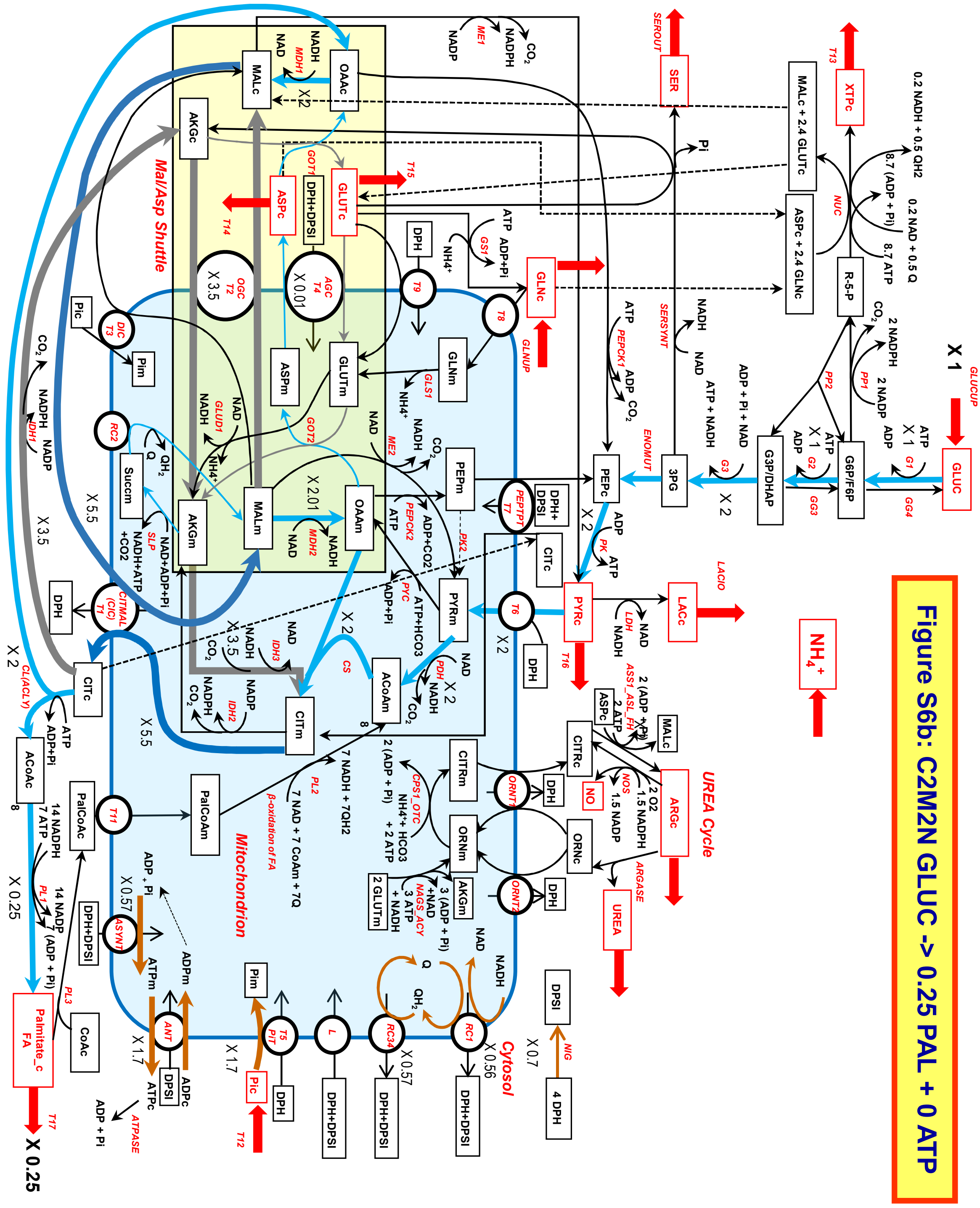


**Figure S6b.** Palmitate synthesis from glucose. Blue and gray arrows represent the main pathways (flux = 2). There is a cycle of MAL (gray and dark blue) to export citrate and a cycle involving (-IDH3) and IDH1 (in gray) to transform NADHm in NADPHc necessary for fatty acids synthesis. Brown arrows depict OXPHOS.

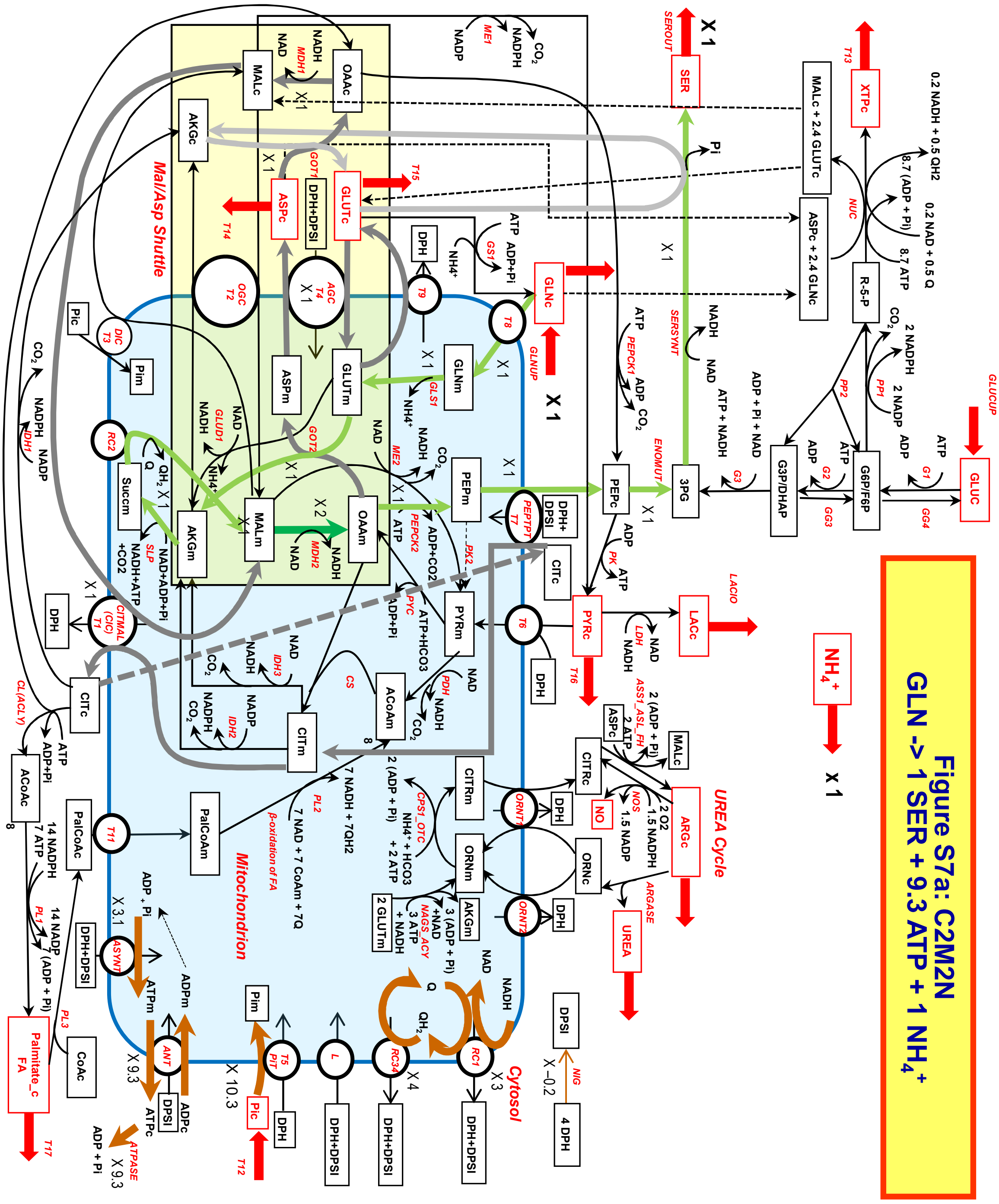


**Figure S7a.** Serine synthesis from glutamine. Green and gray arrows represent the main pathways (flux = 1). The NADHc produced in serine synthesis is reoxidized by the respiratory chain through a shuttle involving the ASP/GLUT carrier (T4), the CIT/MAL carrier (T1), and the T7 antiporter that insure the output of PEP against citrate for 3PG synthesis (in gray). Brown arrows depict OXPHOS.

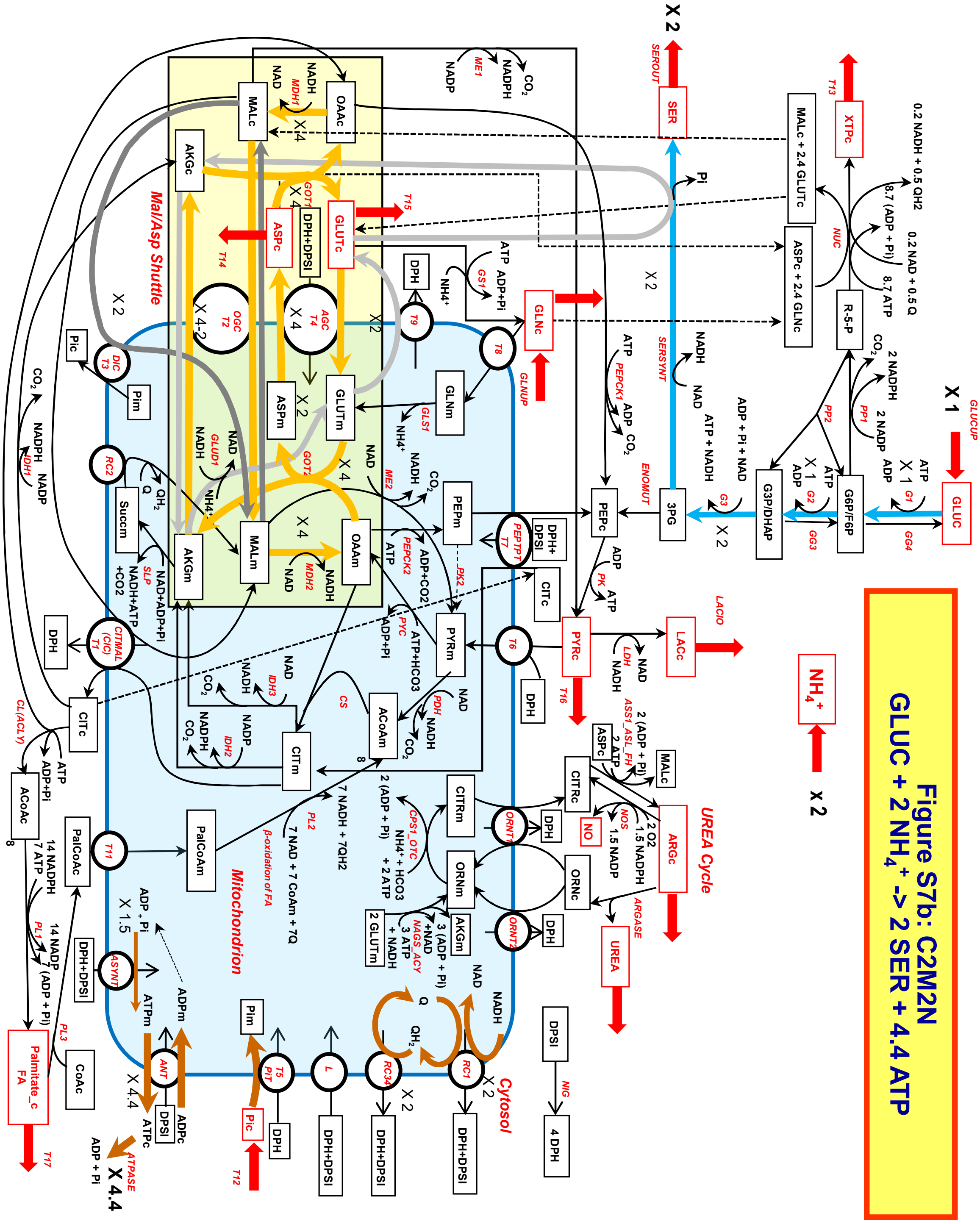


**Figure S7b.** Serine synthesis from glucose. Blue and gray arrows represent the main pathway (flux = 2). Yellow arrows correspond to the malate–aspartate shuttle (flux = 4) to reoxidize NADHc produced by glycolysis and SERSYNT. Brown arrows depict OXPHOS.

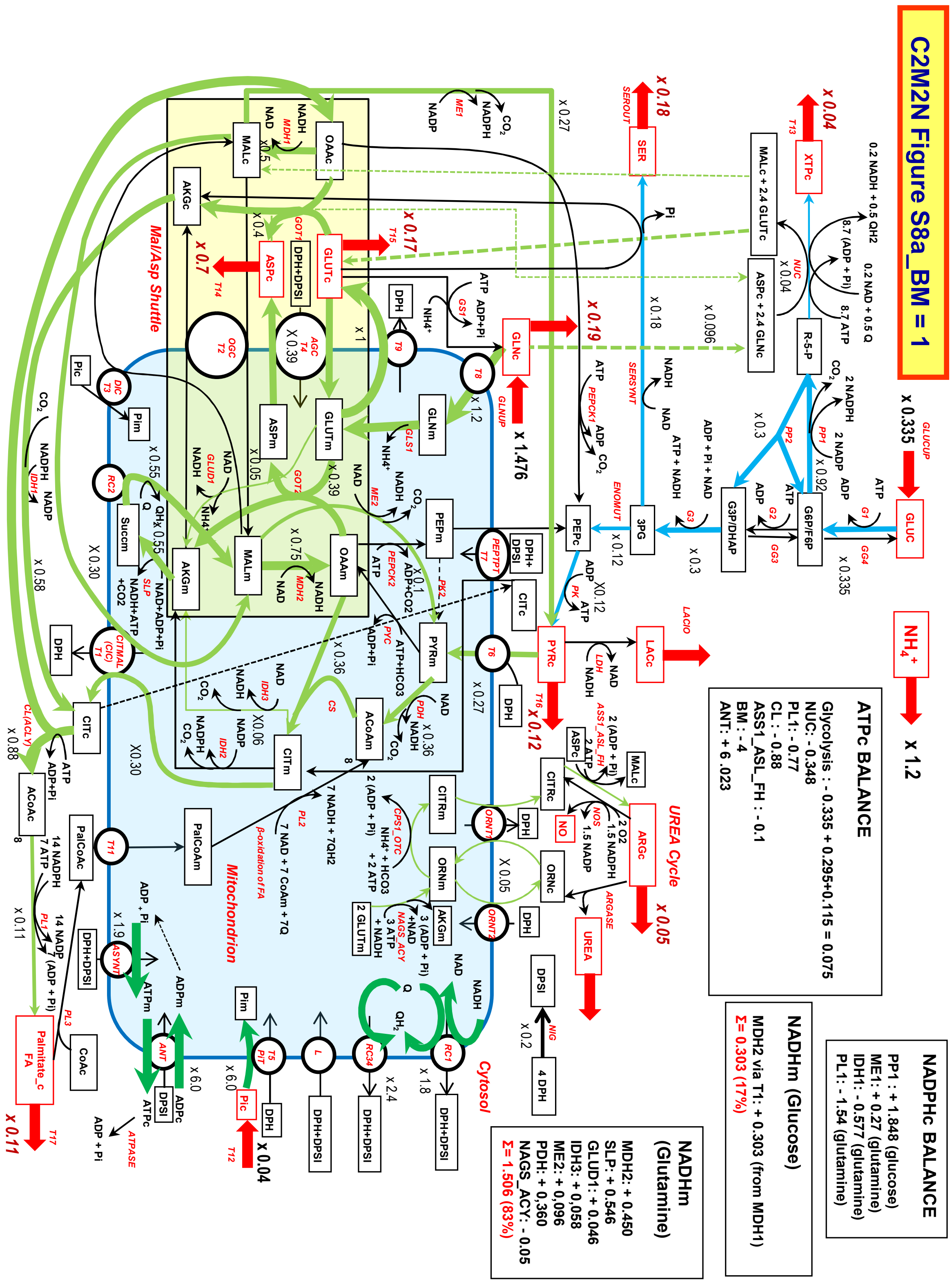


**Figure S8a.** The interplay between glutamine, ammonia, and glucose to sustain cell proliferation. The uptake of glucose and glutamine is free to make 1 arbitrary unit of biomass flux. Glutamine catabolism is in green and glucose catabolism in blue. Nitrogen is incorporated as a large excess of glutamine with release of ammonia. The origin of NADHm (glucose or glutamine) is indicated in the boxes showing that most of mitochondrial ATP synthesis is due to glutamine (it is the reason for dark green respiratory chain). Few ATPc is due to glycolysis (see ATPc balance box). Most of NADP/NADPH cycling is also due to glutamine.

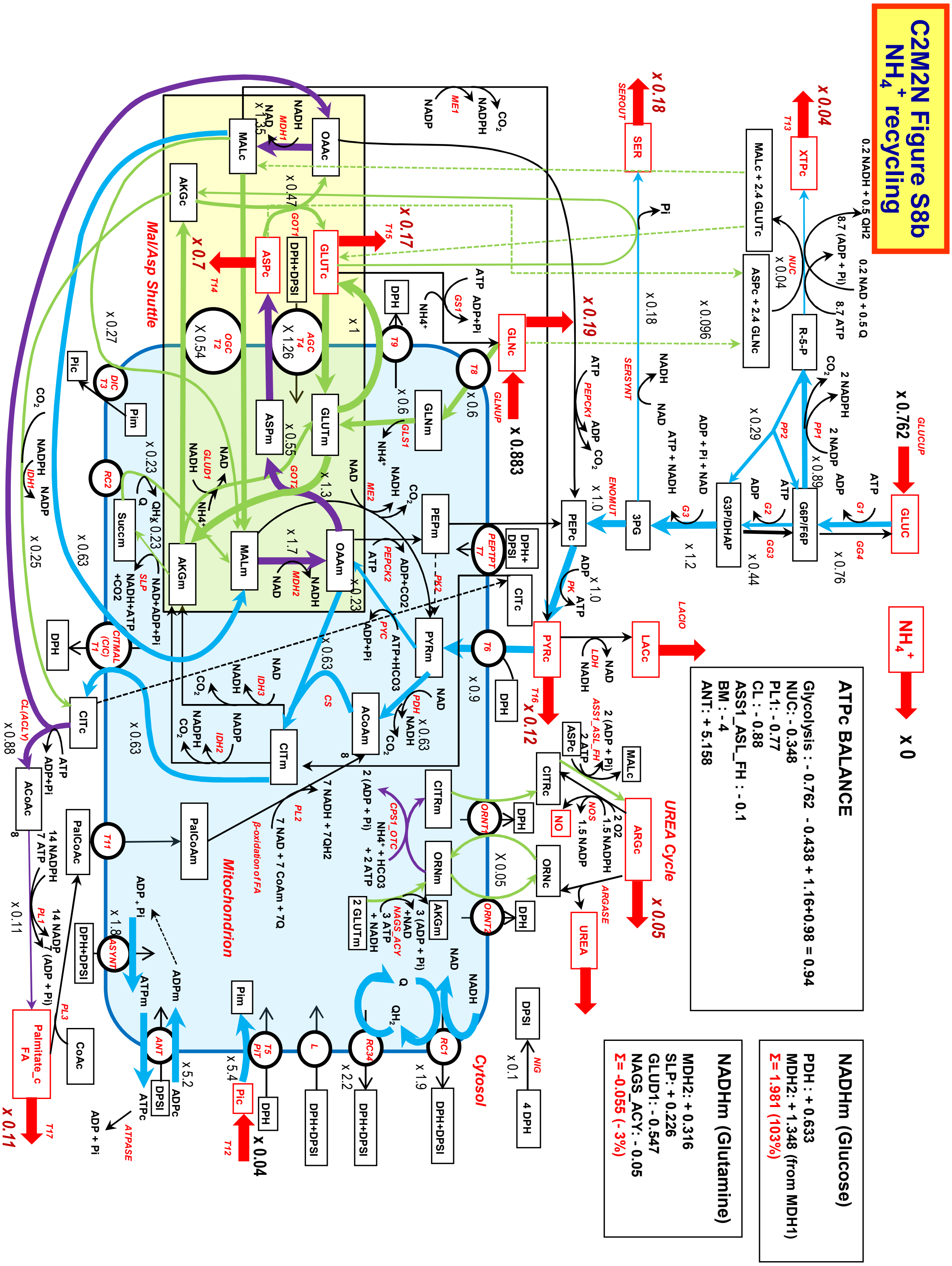


**Figure S8b.** The interplay between glutamine, ammonia and glucose to sustain cell proliferation. This solution depicts a case where ammonia release is cancelled. Ammonia produced by GLS1 is thus stoichiometrically recycled mainly by GLUD1 (glutamate dehydrogenase) in the reverse direction and to a lower extent by carbamyl phosphate synthesis (CPS1 in dark violet) to ultimately give arginine. In this simulation, nearly 100% of ATP is due to glucose (see NADHm and ATP boxes).

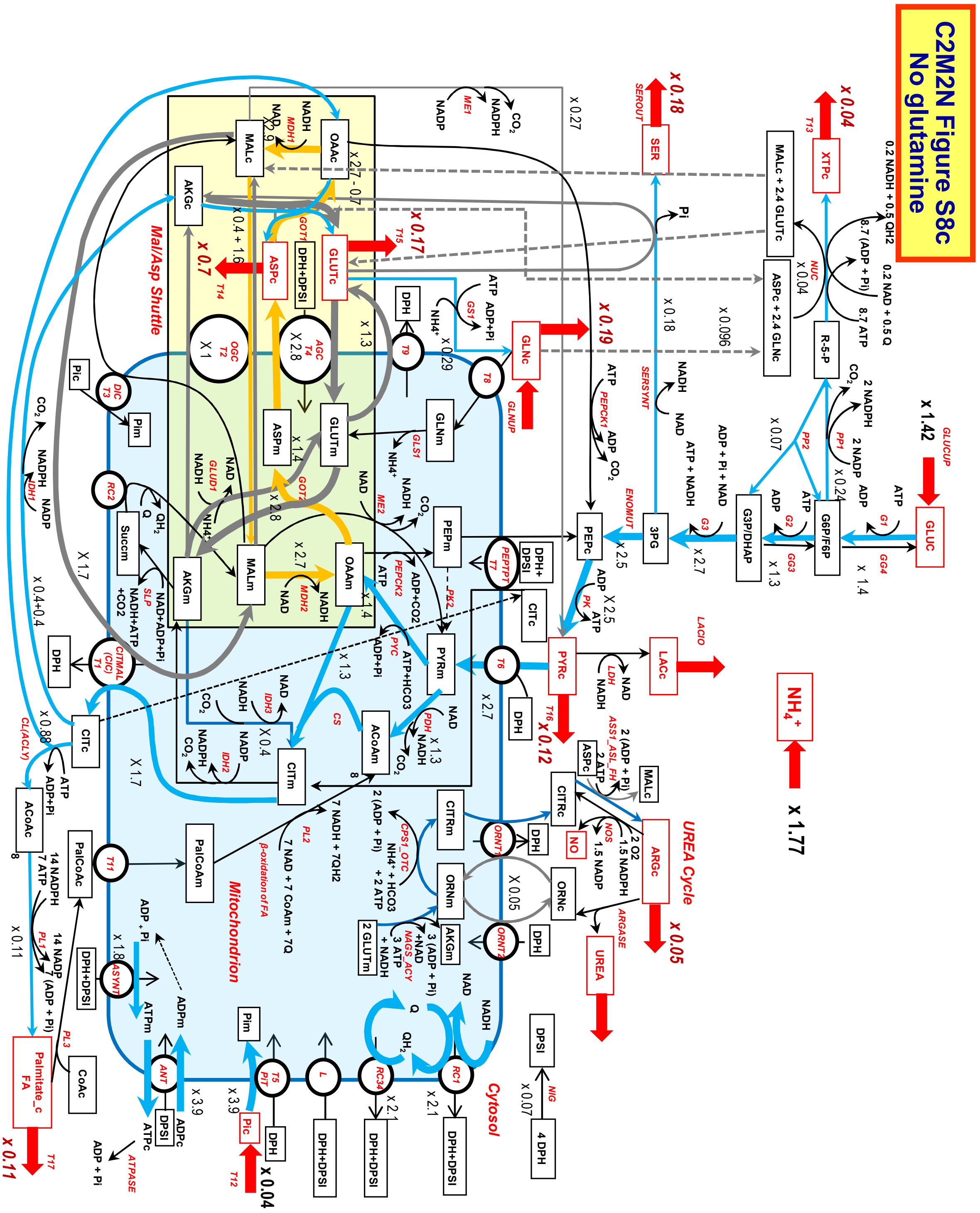


**Figure S8c.** The interplay between ammonia and glucose to sustain cell proliferation. Nitrogen is incorporated as ammonia in glutamate by the reversion of glutamate dehydrogenase (-Glud1) and in glutamine by glutamine synthase (GS1). A small part of $NH_4^+$ is incorporated directly by CPS1 (carbamyl phosphate synthase) for arginine synthesis. Four cycles in yellow (MAS flux = 1.27) and in gray are operating. They are described in Supplementary Materials 2.

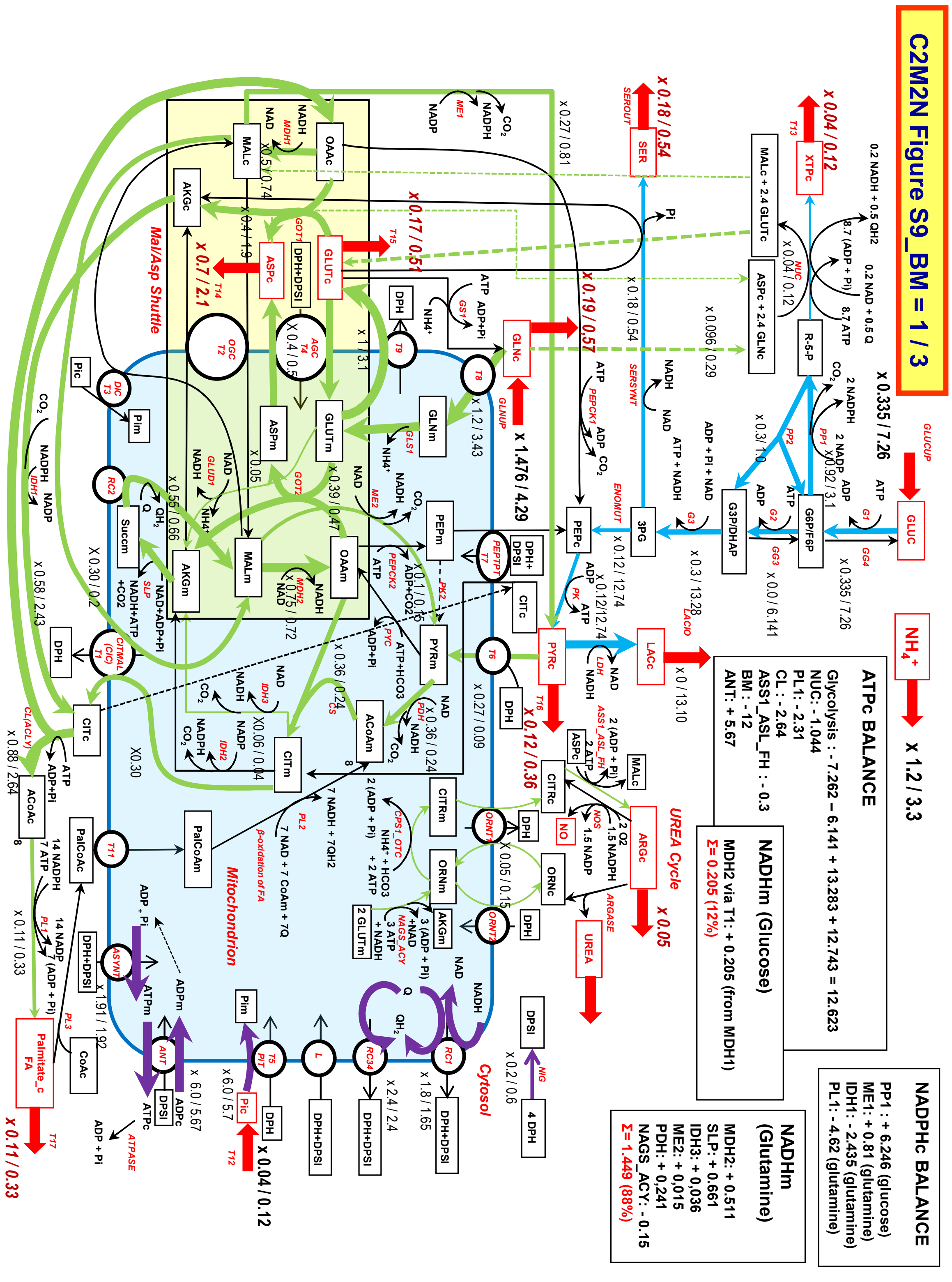


**Figure S9.** The interplay between glutamine, glucose and ammonia to sustain a flux of biomass of 1 or 3 (1/3 on the Figure). The Warburg effect. The fluxes for BM = 1 are identical to the fluxes in Figure S8a. They are kept here for comparison with the fluxes for BM = 3 in order to follow the changes of fluxes in the Warburg effect. In comparison with Figure S8a, the ANT output of mitochondrial ATP is nearly the same (see the ATPc box). The majority of ATPc comes from glycolysis with a large increase in glucose uptake accompanied by a large release of lactate. There is also a nearly 3-fold increase in glutamine uptake corresponding to the 3-fold demand in biomass. The balance boxes are given for the case of a biomass BM = 3
